# The impact of changes in coding on mortality reports using the example of sepsis

**DOI:** 10.1186/s12911-022-01947-x

**Published:** 2022-08-01

**Authors:** Catherine Atkin, Tanya Pankhurst, David McNulty, Ann Keogh, Suzy Gallier, Domenico Pagano, Elizabeth Sapey, Simon Ball

**Affiliations:** 1 PIONEER, HDR-UK Health Data Research Hub in Acute Care, Institute of Inflammation and Ageing, University Hospitals Birmingham NHS Foundation Trust, University of Birmingham, Edgbaston, Birmingham, B15 2GW UK; 2 Renal Medicine, University Hospitals Birmingham NHS Foundation Trust, Birmingham, B15 2GW UK; 3 Department of Health Informatics, University Hospitals Birmingham NHS Foundation Trust, Birmingham, B15 2GW UK; 4 University Hospitals Birmingham NHS Foundation Trust, Birmingham, B15 2GW UK; 5 PIONEER, HDR-UK Health Data Research Hub in Acute Care, University of Birmingham, Birmingham, B15 2GW UK; 6 Cardiac Surgery, University Hospitals Birmingham NHS Foundation Trust, Birmingham, B15 2GW UK; 7 PIONEER, HDR-UK Health Data Research Hub in Acute Care, Institute of Inflammation and Ageing, University of Birmingham, Birmingham, B15 2GW UK; 8 Department of Acute Medicine, University Hospitals Birmingham NHS Foundation Trust, Birmingham, B15 2GW UK; 9 HDR-UK Midlands Site, University Hospitals Birmingham NHS Foundation Trust, Edgbaston, Birmingham, B15 2GW UK

**Keywords:** Morbidity, Clinical coding, Real world data, Epidemiology, Sepsis, Mortality

## Abstract

**Objectives:**

NHS Digital issued new guidance on sepsis coding in April 2017 which was further modified in April 2018. During these timeframes some centres reported increased sepsis associated mortality, whilst others reported reduced mortality, in some cases coincident with specific quality improvement programmes. We hypothesised that changes in reported mortality could not be separated from changes in coding practice.

**Methods:**

Hospital Episode Statistics from the Admitted Patient Care dataset for NHS hospitals in England, from April 2016 to March 2020 were analysed. Admissions of adults with sepsis: an International Classification of Diseases 10 (ICD-10) code associated with the Agency for Healthcare Research and Quality Clinical Classifications Software class ‘Septicaemia (except in labour)’, were assessed. Patient comorbidities were defined by other ICD-10 codes recorded within the admission episode.

**Results:**

1,081,565 hospital episodes with a coded diagnosis of sepsis were studied. After April 2017 there was a significant increase in admission episodes with sepsis coded as the primary reason for admission. There were significant changes in the case-mix of patients with a primary diagnosis of sepsis after April 2017. An analysis of case-mix, hospital and year treated as random effects, defined a small reduction in sepsis associated mortality across England following the first change in coding guidance. No centre specific improvement in outcome could be separated from these random-effects.

**Conclusion:**

Changes in sepsis coding practice altered case-mix and case selection, in ways that varied between centres. This was associated with changes in centre-specific sepsis associated mortality, over time. According to the direction of change these may be interpreted either as requiring local investigation for cause or as supporting coincident changes in clinical practice. A whole system analysis showed that centre specific changes in mortality cannot be separated from system-wide changes. Caution is therefore required when interpreting sepsis outcomes in England, particularly when using single centre studies to inform or support guidance or policy.

**Supplementary Information:**

The online version contains supplementary material available at 10.1186/s12911-022-01947-x.

## Introduction

Sepsis is defined as life-threatening organ dysfunction due to a dysregulated host response to infection [1]. It is an important cause of morbidity and mortality. In 2015, 123,000 cases of sepsis were reported by NHS England (NHSE) to be associated with 36,900 deaths, [2, 3]. Although the reported rate of sepsis has increased over time [4], there is concern that sepsis remains under-recognised, under-diagnosed and under-recorded [5, 6]. Early recognition and treatment is important as there is evidence that this can reduce mortality [7]. Clinical definitions have therefore been extended to include various features, including reduced conscious level and hypoxia as well as hypotension [1].

Initiatives to improve the identification and management of sepsis have been introduced, including public health campaigns raising awareness of the diagnosis [8]. In England, financial incentives were introduced to promote screening for sepsis in emergency departments in 2015 and acute inpatient settings in 2016 [9]. These were followed by recommendations on the use of NEWS2 to screen for sepsis by the Royal College of Physicians (RCP) in 2017 [10], NHSE in 2018 [11] and the National Institute for Health and Care Excellence (NICE) in 2019 [12].

A range of local responses to improve the recognition of sepsis have emerged in primary and secondary care [13, 14], with reports of best-practice identified by NHSE as associated with major reductions in sepsis associated mortality. However, these innovations coincided with national guidance issued to improve the recording of sepsis within the diagnosis [15]. In April 2017 NHS Digital implemented a change in the guidance on how to code a diagnosis of sepsis, with the aim of increasing the identification of sepsis as the primary diagnosis leading to admission. This involved an emphasis on clinical terminology, so that clinicians’ reference to organ specific sepsis, meaning local infection, was more likely to be coded as sepsis. In addition, when conditions such as pneumonia presented with sepsis, there was an emphasis on coding sepsis in the primary position rather than the underlying condition. A further change in guidance was issued from April 2018, advising that this sequencing of conditions should be left to ‘clinical judgement’ [16]. The effect of these changes on Hospital Episode Statistics (HES) derived outcome metrics [17, 18], such as Hospital Standardised Mortality Ratios (HSMR) and Summary Hospital-level Mortality Indicator (SHMI), have been discussed in a briefing document by Dr Foster Intelligence. After the coding change in 2017, an increase in sepsis associated mortality was reported by many hospitals. This triggered further investigation of cause and effect by those hospitals and by the Care Quality Commission (CQC) [19].

We hypothesised that altered coding practice could have affected the sepsis associated mortality rates reported, without changes in actual outcome. This change would confound the interpretation of practice changes introduced during this period. In order to understand this, we undertook a detailed examination of the relationship between altered coding and patient age, type of admission and secondary diagnoses associated with sepsis, [20] or ‘case-mix’. This was with a view to understanding the factors influencing sepsis associated mortality and determining whether centre specific changes in outcome can be differentiated from changes arising from coding practices.

## Methods

Data were extracted from the Hospital Episode Statistics (HES) Admitted Patient Care dataset for the period between April 2016 and March 2020, providing data for 126 NHS acute hospital trusts in England.

Non-identifiable data was accessed and therefore the project did not require specific Human Research Authority (HRA) ethical approval, however, the study was approved by the non-HRA Data Committee at University Hospitals Birmingham NHS Foundation Trust.

Adult patients, aged 18 years or over, with an International Classification of Diseases 10 (ICD-10) code associated with the Agency for Healthcare Research and Quality (AHRQ) Clinical Classifications Software (CCS) class ‘Septicaemia (except in labour)’ recorded in the dominant inpatient episode were identified and is referred to as ‘sepsis’. Patient comorbidities were assessed using the ICD-10 codes recorded within the admission episode, aggregated using the AHRQ CCS categories for ICD-10-CM Diagnoses v2019.1.

Changes to sepsis coding criteria based on recommendations published by NHS Digital were introduced in April 2017 and April 2018. Data were analysed within 12 month sample periods beginning in April each year, providing data for 12 months leading up to the first coding change, the 12 months between coding changes, and the 24 months following the April 2018 coding change.

### Statistical analysis

Data analysis was performed through the Quality and Outcomes Research Unit in conjunction with the Health Informatics Department at University Hospitals Birmingham, using SAS/STAT software version 9.4. Models were constructed including cases with septicaemia coded as the dominant diagnosis. A change in prevalence of a comorbidity was determined by modelling the proportion of patients with the comorbidity before and after the coding changes using a logistic regression model with terms for provider, age and sample period.

Mortality status was obtained from the Office for National Statistics. Hospital mortality was modelled using a generalised linear mixed model. Provider and sample period were treated as random effects. The effects of case-mix, provider and sample period were modelled using the terms identified via backwards elimination logistic regression. Age was represented by a natural cubic spline with six knots placed at even percentiles of the data. Deprivation was represented by Quintiles plus a category for unmatched and missing data. Season was represented by a cyclic linear spline with knots in March (Spring), June (Summer), September (Autumn), and December (Winter).

Backwards elimination logistic regression was performed to identify comorbidities that were associated with death. Comorbidity classes that recorded death, for example ‘Sudden death cause unknown’ were excluded prior to analysis, as were comorbidity classes associated with fewer than 10 deaths in each year, to prevent numeric convergence problems and unstable parameters associated with these small groups. The logistic regression model also included demographic variables (age, sex, ethnicity, index of multiple deprivation), type of admission (emergency or non-emergency), time period, season and provider.

## Results

The number of admissions where sepsis was recorded within the diagnosis is shown in Table 1, increasing from 199,395 between April 2016 and March 2017 (‘2016–2017’) to 312,780 between April 2017 and March 2018 (‘2017–2018’), 291,110 between April 2018 and March 2019 (‘2018–2019’), and 278,270 between April 2019 and March 2020 (‘2019–2020’).

**Table 1:** Number of patients with septicaemia recorded within diagnosis

Time period		Sepsis recorded as primary diagnosis	Sepsis recorded in diagnosis (but not in primary position)	Total number where sepsis recorded in diagnosis	Percentage with sepsis recorded where sepsis in primary position (%)
Apr 2016–Mar 2017		71,400	127,995	199,395	35.8
Apr 2017–Mar 2018		179,760	133,020	312,780	57.5
Apr 2018–Mar 2019		135,140	155,970	291,110	46.4
Apr 2019–Mar 2020		122,870	155,400	278,270	44.2
2016	April	5275	9715	14,990	35.2
	May	5295	10,220	15,515	34.1
	June	5205	10,205	15,410	33.8
	July	5935	10,535	16,470	36.0
	August	5690	10,980	16,670	34.1
	September	5675	10,375	16,050	35.4
	October	5925	10,830	16,755	35.4
	November	5895	10,695	16,590	35.5
	December	6335	11,150	17,485	36.2
2017	January	5915	11,540	17,455	33.9
	February	5920	10,440	16,360	36.2
	March	8335	11,310	19,645	42.4
	April*	13,470	10,480	23,950	56.3
	May	14,850	10,930	25,735	57.7
	June	14,430	11,000	25,430	56.7
	July	15,215	11,060	26,275	57.9
	August	15,270	10,965	26,235	58.2
	September	15,205	10,550	25,755	59.0
	October	15,580	11,115	26,695	58.4
	November	14,940	10,955	25,895	57.7
	December	16,815	11,190	28,005	60.0
2018	January	15,980	12,000	27,980	57.1
	February	13,910	10,550	24,460	56.9
	March	14,140	12,225	26,365	53.6
	April*	11,045	12,450	23,495	47.0
	May	11,440	13,105	24,545	46.6
	June	11,250	12,555	23,805	47.3
	July	12,130	12,980	25,110	48.3
	August	12,070	13,230	25,300	47.7
	September	11,160	12,255	23,415	47.7
	October	11,555	13,480	25,035	46.2
	November	10,980	12,885	23,865	46.0
	December	11,385	13,355	24,740	45.2
2019	January	11,615	14,085	25,700	44.4
	February	10,005	12,510	22,515	44.5
	March	10,505	13,080	23,585	44.2
	April	10,795	13,235	24,030	44.9
	May	10,945	13,005	23,950	45.7
	June	10,660	12,710	23,370	45.6
	July	11,460	13,770	25,230	45.4
	August	11,340	13,195	24,535	46.2
	September	10,550	12,935	23,485	44.9
	October	10,765	13,655	24,420	44.1
	November	10,220	13,415	23,635	43.2
	December	10,515	13,560	24,075	43.7
2020	January	9865	13,455	23,320	42.3
	February	8890	11,760	20,650	43.1
	March	6865	10,705	17,570	39.1

Number of coded admissions for sepsis from hospitals in England, taken from the Hospital Episode Statistics Admitted Patient Care dataset. Changes to coding of septicaemia were introduced in April 2017 and April 2018 (denoted by *). Data has been rounded for reporting. (A reduction in admissions coded for sepsis in March 2020 was associated with a rapid increase in COVID19 admissions and reduction in other admissions, as the pandemic began to take effect in the UK)

In parallel with these changes in the absolute number of admissions in which sepsis was recorded, the proportion in which it was in the primary position increased in 2017–2018 versus 2016–2017 (56.0% vs. 34.6%, *p* < 0.005), after the first change in coding guidance. After the second change in coding guidance in April 2018 this proportion fell to 45.3% but remained significantly higher than in 2016–2017 (45.3% vs. 34.6%, *p* < 0.005). These overall trends were mirrored across different demographic groups, albeit that as age increases the proportion in which sepsis appeared in the primary vs secondary position also increased (Table 2).

**Table 2:** Demographics of patients with a recorded diagnosis of septicaemia

Time period	April 2016–March 2017						April 2017–March 2018						April 2018–March 2019					
Place in coding	Primary		Secondary		Any position		Primary		Secondary		Any position		Primary		Secondary		Any position	
	N	%	N	%	N	%	N	%	N	%	N	%	N	%	N	%	N	%
*Age*																		
18–24	1345	1.9	4330	3.4	5675	2.9	2925	1.6	4885	3.8	7810	2.5	2095	1.6	5695	3.7	7790	2.7
25–44	5315	7.4	14,830	11.6	20,145	10.1	12,070	6.7	16,730	12.6	28,800	9.2	9250	6.8	20,200	13.0	29,450	10.1
45–59	11,055	15.5	20,400	15.9	31,455	15.8	24,545	13.7	21,965	16.5	46,510	14.9	19,685	14.6	25,365	16.3	45,050	15.5
60–69	13,735	19.2	21,985	17.2	35,720	17.9	31,210	17.4	22,800	17.1	54,010	17.3	24,225	17.9	26,470	17.0	50,695	17.4
70–79	17,775	24.9	28,825	22.5	46,600	23.4	45,560	25.3	29,420	22.2	74,980	24.0	35,270	26.1	35,090	22.5	70,360	24.2
80–89	16,935	23.7	28,935	22.6	45,870	23.0	48,420	26.9	28,650	21.5	77,070	24.6	34,360	25.4	33,270	21.3	67,630	23.2
90 +	5240	7.3	8685	6.8	13,925	7.0	15,030	8.4	8565	6.4	23,595	7.5	10,255	7.6	9880	6.3	20,135	6.9
*Sex*																		
Female	35,650	49.9	64,590	50.5	100,240	50.3	87,075	48.4	67,260	50.6	154,335	49.3	63,945	47.3	77,295	49.6	141,240	48.5
Male	35,755	50.1	63,395	49.5	99,150	49.7	92,690	51.6	65,760	49.4	158,450	50.7	71,200	52.7	78,665	50.4	149,865	51.5
*Ethnicity*																		
White	61,045	85.5	106,365	83.1	167,410	84.0	154,015	85.7	108,080	81.3	262,095	83.8	113,815	84.2	125,950	80.8	239,765	82.4
Asian/Asian British	2835	4.0	6580	5.1	9415	4.7	7235	4.0	6885	5.2	14,120	4.5	6005	4.4	8700	5.6	14,705	5.1
Black/Black British	1290	1.8	3050	2.4	4340	2.2	2780	1.6	3610	2.7	6390	2.0	2565	1.9	4075	2.6	6640	2.3
Mixed	365	0.4	600	0.5	865	0.4	700	0.4	705	0.5	1450	0.5	585	0.4	880	0.6	1465	0.5
Other ethnic groups	4785	6.7	8670	6.8	13,455	6.7	11,730	6.5	10,230	7.7	21,960	7.0	9450	7.0	12,485	8.0	21,935	7.5
Unknown	1185	1.7	2720	2.1	3905	2.0	3310	1.8	3505	2.6	6815	2.2	2720	2.0	3870	2.5	6590	2.3
*IMD quintile*																		
1 (lowest)	13,005	18.2	19,745	15.4	32,750	16.4	30,935	17.2	20,705	15.6	51,640	16.5	22,970	17.0	25,240	16.2	48,210	16.6
2	14,220	19.9	23,055	18.0	37,275	18.7	34,710	19.3	23,920	18.0	58,630	18.7	25,990	19.2	29,120	18.7	55,110	18.9
3	14,770	20.7	25,185	19.7	39,955	20.0	36,650	20.4	26,090	19.6	62,740	20.1	27,200	20.1	30,640	19.7	57,840	19.9
4	14,530	20.4	27,495	21.5	42,025	21.1	37,280	20.7	28,860	21.7	66,140	21.2	28,090	20.8	32,890	21.1	60,980	21.0
5 (highest)	14,355	20.1	31,170	24.4	45,525	22.8	38,710	21.5	32,020	24.1	70,730	22.6	29,685	22.0	36,000	23.1	65,685	22.6
Unknown	520	0.7	1335	1.0	1855	0.9	1485	0.8	1425	1.1	2910	0.9	1205	0.9	2075	1.3	3280	1.1
*Admission type*																		
Emergency	68,210	95.5	105,780	82.7	173,990	87.3	173,615	96.6	104,340	78.4	277,955	88.9	130,315	96.4	123,640	79.3	253,950	87.2
Non-emergency	3190	4.5	22,205	17.4	25,395	12.7	6150	3.4	28,680	21.6	34,830	11.1	4835	3.6	32,325	20.7	37,160	12.8

Time period	April 2019–March 2020						Odds ratio for sepsis recorded in primary position (compared to 2016/17)		
Place in coding	Primary		Secondary		Any position		2017/18	2018/19	2019/20
	N	%	N	%	N	%	OR (95% CI)	OR (95% CI)	OR (95% CI)
*Age*									
18–24	1770	1.4	5150	3.3	6920	2.5	1.93 (1.78–2.08)	1.18 (1.09–1.28)	1.11 (1.02–1.20)
25–44	8265	6.7	19,885	12.8	28,150	10.1	2.01 (1.93–2.09)	1.28 (1.23–1.33)	1.16 (1.11–1.20)
45–59	17,425	14.2	25,110	16.2	42,535	15.3	2.06 (2.00–2.12)	1.43 (1.39–1.48)	1.28 (1.24–1.32)
60–69	21,345	17.4	26,100	16.8	47,445	17.1	2.19 (2.13–2.25)	1.47 (1.43–1.51)	1.31 (1.27–1.35)
70–79	32,865	26.8	36,000	23.2	68,865	24.8	2.51 (2.45–2.57)	1.63 (1.59–1.67)	1.48 (1.45–1.52)
80–89	31,730	25.8	33,240	21.4	64,970	23.4	2.89 (2.82–2.96)	1.76 (1.72–1.81)	1.63 (1.59–1.67)
90 +	9480	7.7	9915	6.4	19,395	7.0	2.91 (2.79–3.04)	1.72 (1.65–1.80)	1.58 (1.52–1.66)
*Sex*									
Female	57,530	46.8	76,975	49.5	134,505	48.3	2.35 (2.31–2.38)	1.50 (1.47–1.52)	1.35 (1.33–1.38)
Male	65,355	53.2	78,425	50.5	143,780	51.7	2.50 (2.46–2.54)	1.61 (1.58–1.63)	1.48 (1.45–1.50)
*Ethnicity*									
White	102,425	84.4	124,150	79.9	226,575	81.4	2.48 (2.45–2.51)	1.57 (1.55–1.59)	1.44 (1.42–1.46)
Asian/Asian British	5265	4.3	8960	5.8	14,225	5.1	2.44 (2.31–2.58)	1.60 (1.52–1.69)	1.36 (1.29–1.44)
Black/Black British	2465	2.0	3810	2.5	6275	2.3	1.82 (1.67–1.98)	1.49 (1.37–1.62)	1.53 (1.41–1.66)
Mixed	540	0.4	895	0.6	1435	0.5	1.63 (1.38–1.93)	1.09 (0.92–1.29)	0.99 (0.84–1.17)
Other ethnic groups	9250	7.5	13,570	8.7	22,820	8.2	2.08 (1.98–2.17)	1.37 (1.31–1.43)	1.24 (1.18–1.29)
Unknown	2930	2.4	4015	2.6	6945	2.5	2.17 (1.99–2.36)	1.61 (1.48–1.75)	1.68 (1.54–1.82)
*IMD quintile*									
1 (lowest)	20,590	16.8	24,700	15.9	45,290	16.3	2.27 (2.20–2.33)	1.38 (1.34–1.42)	1.27 (1.23–1.30)
2	23,770	19.3	28,480	18.3	52,250	18.8	2.35 (2.29–2.42)	1.45 (1.41–1.49)	1.35 (1.32–1.39)
3	24,815	20.2	30,990	19.9	55,805	20.1	2.40 (2.33–2.46)	1.51 (1.47–1.55)	1.37 (1.33–1.40)
4	25,110	20.4	32,520	20.9	57,630	20.7	2.44 (2.38–2.51)	1.62 (1.58–1.66)	1.46 (1.42–1.50)
5 (highest)	26,590	21.6	35,560	22.9	62,150	22.3	2.63 (2.56–2.69)	1.79 (1.75–1.84)	1.62 (1.58–1.67)
Unknown	2005	1.6	3145	2.0	5150	1.9	2.68 (2.36–3.03)	1.49 (1.32–1.69)	1.64 (1.46–1.84)
*Admission type*									
Emergency	118,710	96.9	122,800	79.0	241,510	86.8	2.58 (2.55–2.61)	1.63 (1.61–1.65)	1.50 (1.48–1.52)
Non-emergency	4175	3.4	32,600	21.0	36,775	13.2	1.49 (1.43–1.56)	1.04 (0.99–1.09)	0.89 (0.85–0.94)

*IMD* Index of Multiple Deprivation quintile. Percentages refer to percentage within the column; row percentages not shown. Odds ratio for recording of sepsis in primary position for those where sepsis recorded in diagnosis, compared to April 2016–March 2017. Data has been rounded for reporting

**Fig. 1:**
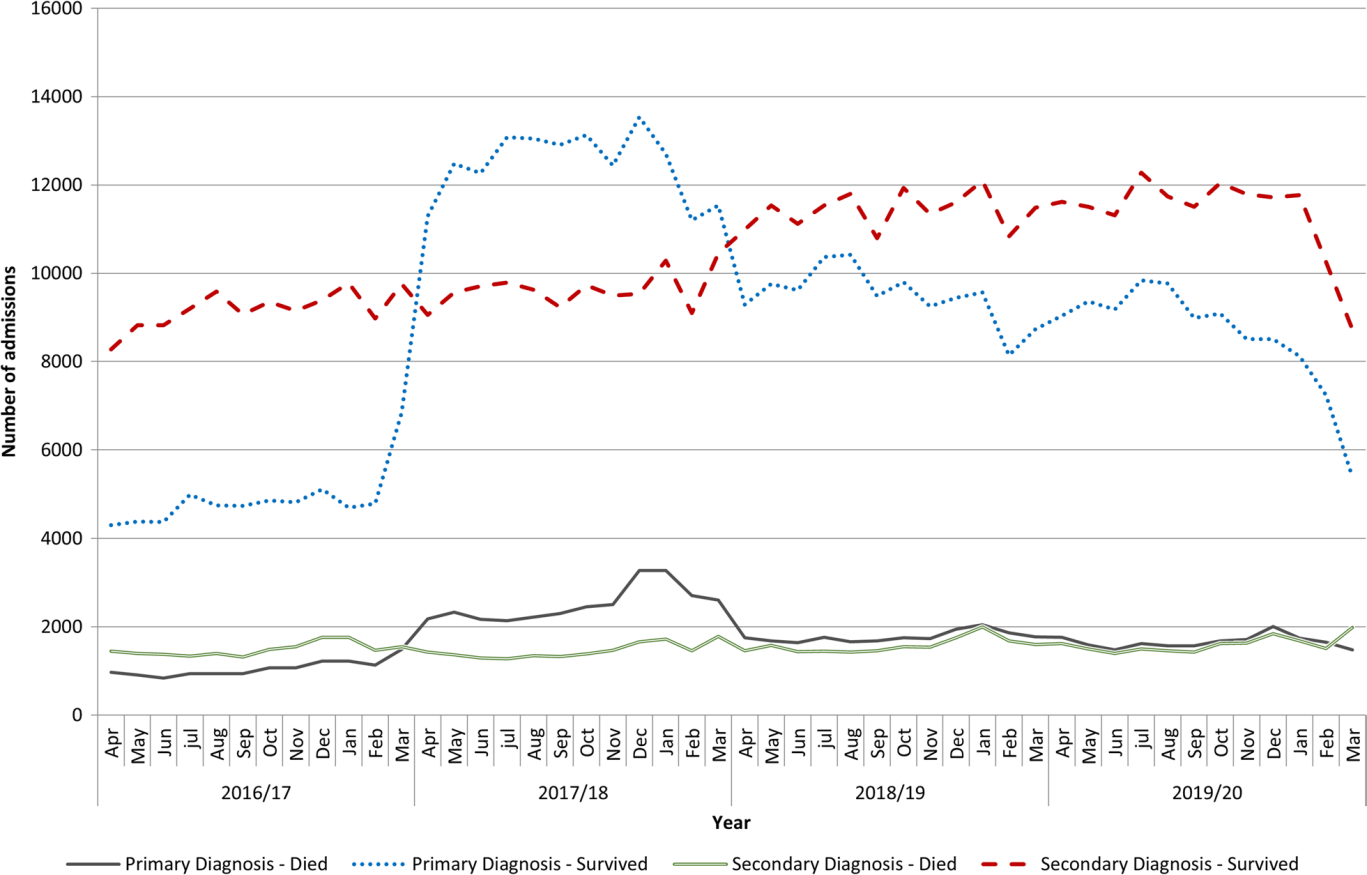
Outcome of admission episodes with a diagnosis of septicaemia. Legend. Number of admissions shown for patients with septicaemia as primary diagnosis, or as secondary diagnosis, for survivors and for those who died, presented by month. Data from Hospital Episode Statistics (HES) Admitted Patient Care dataset

Inpatient mortality following admission with a diagnosis of sepsis is summarised in Fig. 1. Inpatient mortality associated with sepsis coded in the primary position fell from 17.8% (2016–2017) to 16.8% (2017–2018), 15.7% (2018–2019) and 16.2% (2019–2020). However, changes in coding practice were also associated with significant changes in comorbidities coded in the secondary positions, shown in Additional file 1: Table S1 and summarised in Table 3. Thus for 2017–2018 compared to 2016–2017, 84 diagnostic codes were more common and 27 less common in patients with sepsis in the primary position. Of those that were more common, 53 were associated with increased mortality and 31 with decreased mortality. Of those that were less common, 20 were associated with increased mortality and 7 with decreased mortality.

**Table 3:** Prevalence of comorbidities in patients with a primary diagnosis of septicaemia

Prevalence of comorbidity	Effect of comorbidity on mortality		Total
	Increased	Decreased	
Increased	53^a^	31^b^	84
Unchanged	9	6	15
Decreased	20^b^	7^a^	27
Total	82	44	

Change in prevalence of comorbidities in patients with a primary diagnosis of septicaemia, comparing April 2016–March 2017 to April 2017–March 2018

^a^Change may worsen mortality rate

^b^Change may benefit mortality rate

As there was a change in the case-mix of patients with sepsis in the primary position, case-mix adjusted log odds of death were calculated from the mortality risk of comorbidities. The median log odds of death accounting for all coded comorbidities increased from − 2.21 (2016–2017) to − 2.10 (2017–2018), − 2.15 (2018–2019) and − 2.11 (2019–2020). There was therefore a small but significant increase in the calculated mortality risk of the population with sepsis in the primary position.

**Fig. 2:**
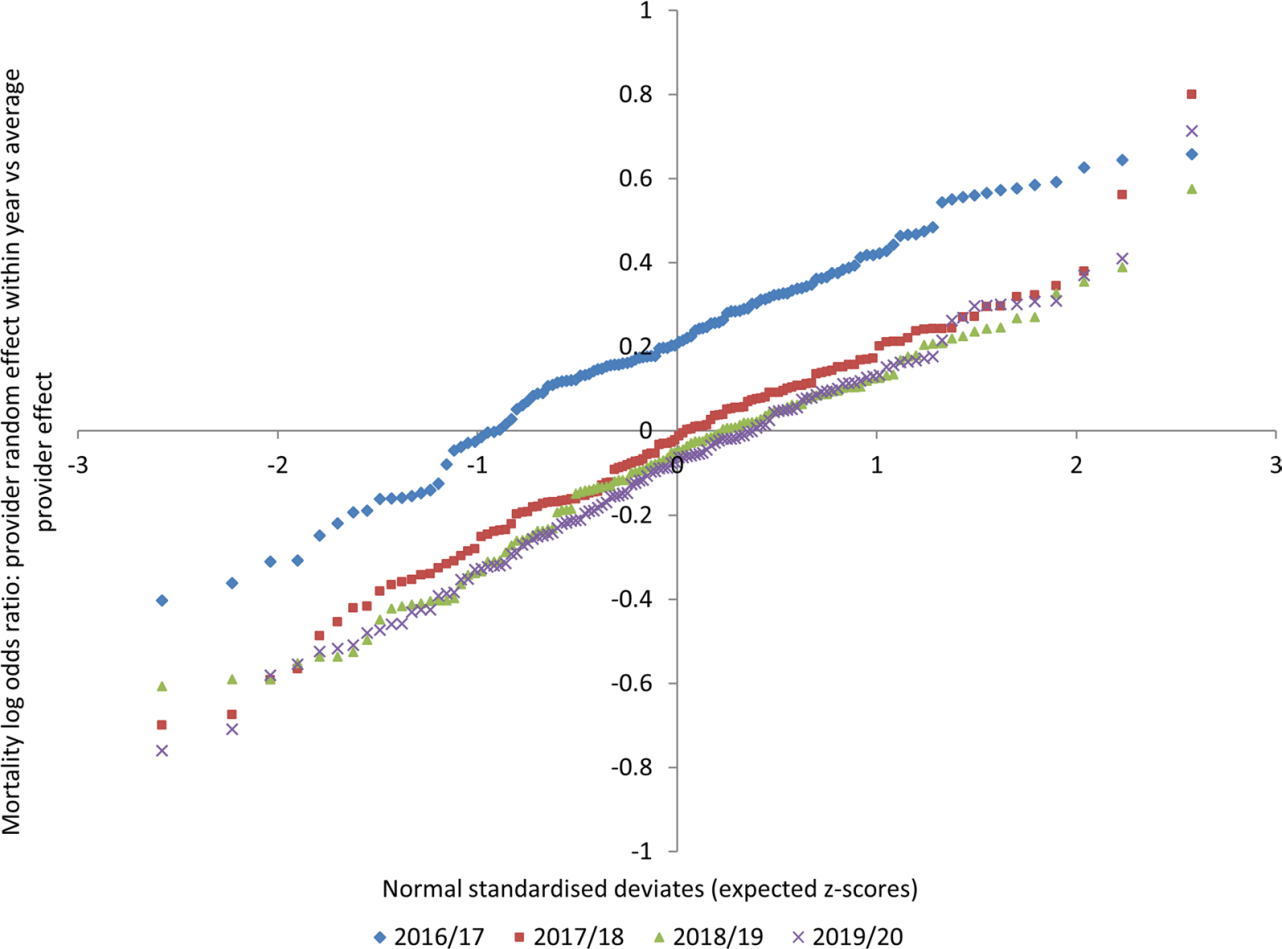
Hospital random effects model of case-mix adjusted mortality

Figure 2 presents the findings of the mixed model in which provider and year were treated as random effects. The observed sepsis associated mortality (the log odds ratio for the provider in the year vs the average provider across all years) is plotted against the expected mortality (the within year normal standardised deviates of the case-mix adjusted predicted mortality). In this analysis, mortality falls from 2016–2017 to 2017–2018, and reduces a little further in the subsequent 2 years. This reduction in mortality occurs despite the adjusted mortality risk derived from the coded comorbidities increasing. The shift in mortality is consistent across all but one centre, which exhibited higher than expected mortality across all three years following the coding change. All other centres form a continuous distribution across the years studied; there were no other outlying centres in which the observed mortality significantly differed from that expected in 2018–2019 and 2019–2020.

## Discussion

The recognition and response to sepsis is of concern to patients and healthcare professionals, since it is a significant cause of morbidity and mortality [2, 3]. NHSE have used various means to improve the recognition and treatment of sepsis, most recently mandating the use of NEWS2 in acute hospital trusts to screen for sepsis [10, 11]. The effect of such a screening strategy is poorly understood [21], nevertheless a range of centre specific quality improvement programmes have been described and endorsed by NHSE, in ways that appear to link process change to outcome, resulting in statements such as: *‘Hundreds of lives saved through new tech to spot sepsis’* [22]. This interpretation reflects the results of interrupted time series, during a period in which NHS coding guidelines for sepsis have changed twice, in April 2017 and April 2018. The effects of these changes on standardised measures of hospital mortality have been recognised. Our analysis set out to understand their effects on interpretation of centre specific changes in sepsis associated mortality, analysing Hospital Episode Statistics from 2016 to 2020.

As intended, in April 2017 there was a substantial increase in the number of patients in which sepsis was defined as the primary diagnosis. There was a coincident change in the case-mix which would have been expected to increase mortality associated with a primary diagnosis of sepsis. There were for example, 111 comorbidities associated with a change in sepsis associated mortality, whose prevalence changed following the introduction of the new coding recommendation. Comparing sepsis mortality across these time periods must therefore consider that this altered case-mix will itself impact, and increase, expected mortality, as the population now defined as sepsis has altered. This alteration in comorbidities contributes to complexity in the interpretation of changes in sepsis associated mortality within and between individual centres, in which there may be different population patterns of comorbidity and differences in coding practice, accentuated at times of change in coding as well as clinical practice [17, 19].

The mixed methods analysis performed here separates the effects of case-mix and centre, adjusting for the changes in case-mix that influence sepsis associated mortality, facilitating comparison of effects related to centre to expected distributions. This showed that variation in mortality between centres follows parallel distributions before and after coding changes, suggesting a systemic change that affected sepsis associated mortality across centres. This presentation focuses on observations which are inconsistent with expectations, identifying just one centre where reported mortality is higher than expected in 2018–2019 and 2019–2020. No centre was identified in which sepsis associated mortality was significantly better than expected. Although this does not preclude the possibility that specific interventions have influenced outcomes in individual centres, these would not be of sufficient magnitude to allow their identification within the observed level of random variation, including unpredictable changes in the application of coding guidance. After April 2018, the sequencing of conditions in episodes in which sepsis appears was left to ‘clinical judgement’, so that interactions between coder and clinician further influences local coding. This may itself be affected by conduits for that interaction, including the electronic healthcare record, and the emergence of local applications of specific terminologies, particularly in the context of local sepsis awareness campaigns. These are some of the potential contributors to unaccounted for inter-centre variation in coding for sepsis.

Although not formally proven, it is extremely likely that the shift in mortality from April 2017 is consequent upon systematic increases in coding for sepsis in the primary position, in patients with a lower mortality risk, that is to say with less severe disease, rather than being due to changes in clinical practice. This would be an expected consequence of a policy that sets out to increase coding for sepsis. Furthermore, there was no universal intervention introduced in April 2017 that would otherwise account for such a consistent change towards lower sepsis associated mortality across centres (with one centre excepted). Caution should therefore be exercised when interpreting the outcomes of interventions introduced from 2016 to 2018 in particular. This situation has now been further complicated by the emergence of COVID19.

We set out to understand whether reasonable conclusions could be made regarding the benefits of specific interventions, during a period of rapid change. Our findings illustrate the general issue of comparing outcomes, on the background of changing data definitions and standards over time and across systems [1, 23]. Use of coded data to provide longitudinal comparison and monitoring of outcomes, including mortality, is reliable only where definitions have remained consistent. Change in nationally reported mortality rates for specific diseases has been demonstrated previously following the introduction of new coding systems [24, 25]. The specific case of sepsis is important, given that the benefits of national policy and resulting organisational and individual behaviours, remain unproven [12, 26]. This needs to be considered by individual centres and regulators responding to adverse changes in sepsis associated mortality, as well as policy makers interpreting changes in outcome. This is not simply a question of case-mix but of case selection, changes in phenotype that are not captured within the case-mix.

Assessment of the effects of process or policy change could be improved, independent of the effects of coding, by using underlying data from electronic healthcare records, to consistently define the phenotype of interest. Previous research suggests that utilising clinical data from electronic healthcare records to monitor sepsis may provide more accurate estimates of sepsis incidence and its associated mortality in comparison to coded diagnoses [27]. Also, the NHS would be well placed to implement a systematic approach to the evaluation of interventions in the electronic environment, through cluster randomised studies; an approach well illustrated in a recent study of the automated identification of adults at risk of deterioration in hospitals in Northern California [28]. A more robust approach to the evaluation of practice than longitudinal analysis post policy change.

Our study relies on clinical coding which is recognised to under-report relevant comorbidities [29], vary between hospitals [30] and between diagnoses [31]. This does not alter our conclusions, rather is the reason to account for random effects in our analysis. The findings do not invalidate evaluations of interventions reporting improvements in process.

## Conclusion

Changes in the coding of sepsis from medical records in England altered the case-mix and case-selection of patients, altering the expected mortality rate in patients where sepsis was recorded as a primary diagnosis. These changes resulted in systematic and random effects which impact upon the interpretation of centre specific mortality rates over time. This is therefore relevant to local quality management and improvement.

Although this focuses on sepsis, the principle applies to other fields where coding practice is subject to intended change or unrecognised drift. This must be considered in determining any clinical response recommendations arising from uncontrolled evidence.

## Supplementary Information

**Additional file 1.** Comorbidity codes associated with an altered risk of mortality, where prevalence of the comorbidity code changedin those with a primary diagnosis of septicaemia.

## Data Availability

The datasets used and/or analysed during the current study are available from the corresponding author on reasonable request.
