# Supplementary material for: The impact of changes in coding on mortality reports using the example of sepsis
Source: BMC Med Inform Decis Mak. 2022 Aug 1;22:204. doi: 10.1186/s12911-022-01947-x (PMC9341053; doi:10.1186/s12911-022-01947-x)
Supplement: Supplementary file 1 — Additional file 1. Comorbidity codes associated with an altered risk of mortality, where prevalence of the comorbidity code changedin those with a primary diagnosis of septicaemia. [file 12911_2022_1947_MOESM1_ESM.docx]

| **Increased prevalence, increased mortality** | **Increased prevalence, decreased mortality** | **Decreased prevalence, increased mortality** | **Decreased prevalence, decreased mortality** |
| --- | --- | --- | --- |
| L1 3 - Endocrine; nutritional; and metabolic diseases and immunity disorders  L1 5 - Mental Illness  L1 8 - Diseases of the respiratory system  L1 9 - Diseases of the digestive system  L1 16 - Injury and poisoning  L2 6.1 - Central nervous system infection  L2 6.2 - Hereditary and degenerative nervous system conditions  L2 7.2 - Diseases of the heart  L2 7.4 - Diseases of arteries; arterioles; and capillaries  L2 10.1 - Diseases of the urinary system  CCS 32 - Cancer of bladder  CCS 36 - Cancer of thyroid  CCS 51 - Other endocrine disorders  CCS 52 - Nutritional deficiencies  CCS 55 - Fluid and electrolyte disorders  CCS 58 - Other nutritional; endocrine; and metabolic disorders  CCS 652 - Attention-deficit conduct and disruptive behavior disorders  CCS 653 - Delirium dementia and amnestic and other cognitive disorders  CCS 654 - Developmental disorders  CCS 660 - Alcohol-related disorders  CCS 79 - Parkinson`s disease  CCS 80 - Multiple sclerosis  CCS 82 - Paralysis  CCS 83 - Epilepsy; convulsions  CCS 97 - Peri-; endo-; and myocarditis; cardiomyopathy (except that caused by tuberculosis or sexually transmitted disease)  CCS 100 - Acute myocardial infarction  CCS 103 - Pulmonary heart disease  CCS 105 - Conduction disorders  CCS 106 - Cardiac dysrhythmias  CCS 107 - Cardiac arrest and ventricular fibrillation  CCS 108 - Congestive heart failure; nonhypertensive  CCS 111 - Other and ill-defined cerebrovascular disease  CCS 114 - Peripheral and visceral atherosclerosis  CCS 116 - Aortic and peripheral arterial embolism or thrombosis  CCS 122 - Pneumonia (except that caused by tuberculosis or sexually transmitted disease)  CCS 129 - Aspiration pneumonitis; food/vomitus  CCS 130 - Pleurisy; pneumothorax; pulmonary collapse  CCS 131 - Respiratory failure; insufficiency; arrest (adult)  CCS 132 - Lung disease due to external agents  CCS 141 - Other disorders of stomach and duodenum  CCS 144 - Regional enteritis and ulcerative colitis  CCS 145 - Intestinal obstruction without hernia  CCS 148 - Peritonitis and intestinal abscess  CCS 152 - Pancreatic disorders (not diabetes)  CCS 156 - Nephritis; nephrosis; renal sclerosis  CCS 157 - Acute and unspecified renal failure  CCS 158 - Chronic kidney disease  CCS 173 - Menopausal disorders  CCS 209 - Other acquired deformities  CCS 216 - Nervous system congenital anomalies  CCS 217 - Other congenital anomalies  CCS 244 - Other injuries and conditions due to external causes  CCS 252 - Malaise and fatigue | L1 14 - Congenital anomalies  L2 2.16 - Benign neoplasms  L2 9.4 - Upper gastrointestinal disorders  L2 9.6 - Lower gastrointestinal disorders  L2 10.3 - Diseases of female genital organs  CCS 3 - Bacterial infection; unspecified site  CCS 48 - Thyroid disorders  CCS 49 - Diabetes mellitus without complication  CCS 50 - Diabetes mellitus with complications  CCS 53 - Disorders of lipid metabolism  CCS 54 - Gout and other crystal arthropathies  CCS 657 - Mood disorders  CCS 662 - Suicide and intentional self-inflicted injury  CCS 67 - Substance-related mental disorders  CCS 93 - Conditions associated with dizziness or vertigo  CCS 98 - Essential hypertension  CCS 112 - Transient cerebral ischemia  CCS 124 - Acute and chronic tonsillitis  CCS 128 - Asthma  CCS 134 - Other upper respiratory disease  CCS 149 - Biliary tract disease  CCS 159 - Urinary tract infections  CCS 160 - Calculus of urinary tract  CCS 161 - Other diseases of kidney and ureters  CCS 163 - Genitourinary symptoms and ill-defined conditions  CCS 164 - Hyperplasia of prostate  CCS 203 - Osteoarthritis  CCS 205 - Spondylosis; intervertebral disc disorders; other back problems  CCS 208 - Acquired foot deformities  CCS 211 - Other connective tissue disease  CCS 253 - Allergic reactions | L1 2 - Neoplasms  L2 1.3 - Viral infection  L2 2.0 - Cancer of lymphatic and hematopoietic tissue  L2 2.2 - Other gastrointestinal cancer  L2 4.1 - Anemia  CCS 11 - Cancer of head and neck  CCS 19 - Cancer of bronchus; lung  CCS 26 - Cancer of cervix  CCS 28 - Cancer of other female genital organs  CCS 33 - Cancer of kidney and renal pelvis  CCS 39 - Leukemias  CCS 42 - Secondary malignancies  CCS 43 - Malignant neoplasm without specification of site  CCS 44 - Neoplasms of unspecified nature or uncertain behavior  CCS 62 - Coagulation and hemorrhagic disorders  CCS 85 - Coma; stupor; and brain damage  CCS 153 - Gastrointestinal hemorrhage  CCS 226 - Fracture of neck of femur (hip)  CCS 249 - Shock  CCS 251 - Abdominal pain | L1 4 - Diseases of the blood and blood-forming organs  L2 16.11 - Poisoning  CCS 24 - Cancer of breast  CCS 63 - Diseases of white blood cells  CCS 102 - Nonspecific chest pain  CCS 120 - Hemorrhoids  CCS 246 - Fever of unknown origin |

**Appendix Table 1:** Comorbidity codes associated with an altered risk of mortality, where prevalence of the comorbidity code changed in those with a primary diagnosis of septicaemia comparing the 12 months before and after the introduction of new coding recommendations in April 2017
